# Hyaluronan-Cyclodextrin Conjugates as Doxorubicin Delivery Systems

**DOI:** 10.3390/pharmaceutics15020374

**Published:** 2023-01-21

**Authors:** Noemi Bognanni, Maurizio Viale, Luana La Piana, Simone Strano, Rosaria Gangemi, Cinzia Lombardo, Maria Teresa Cambria, Graziella Vecchio

**Affiliations:** 1 Dipartimento di Scienze Chimiche, Università degli Studi di Catania, Viale A. Doria 6, 95125 Catania, Italy; 2 UOC Bioterapie, IRCCS Ospedale Policlinico San Martino, Largo R. Benzi 10, 16132 Genova, Italy; 3 Dipartimento di Scienze Biomediche e Biotecnologiche, Sezione di Biochimica Medica, Università degli Studi di Catania, Via S. Sofia 97, 95125 Catania, Italy

**Keywords:** cyclodextrins, doxorubicin, CD44, hyaluronic acid, hyaluronan-cyclodextrin conjugate, nanoparticles, nanomedicine, SK-N-SH

## Abstract

In the last years, nanoparticles based on cyclodextrins have been widely investigated for the delivery of anticancer drugs. In this work, we synthesized nanoparticles with a hyaluronic acid backbone functionalized with cyclodextrins under green conditions. We functionalized hyaluronic acid with two different molecular weights (about 11 kDa and 45 kDa) to compare their behavior as doxorubicin delivery systems. We found that the new hyaluronan-cyclodextrin conjugates increased the water solubility of doxorubicin. Moreover, we tested the antiproliferative activity of doxorubicin in the presence of the new cyclodextrin polymers in SK-N-SH and SK-N-SH-PMA (over-expressing CD44 receptor) cancer cells. We found that hyaluronan-cyclodextrin conjugates improved the uptake and antiproliferative activity of doxorubicin in the SK-N-SH-PMA compared to the SK-N-SH cell line at the ratio 8/1 doxorubicin/polymer. Notably, the system based on hyaluronan (45 kDa) was more effective as a drug carrier and significantly reduced the IC_50_ value of doxorubicin by about 56%. We also found that hyaluronic acid polymers determined an improved antiproliferative activity of doxorubicin (IC_50_ values are on average reduced by about 70% of free DOXO) in both cell lines at the ratio 16/1 doxorubicin/polymer.

## 1. Introduction

In the last years, the development of nanoparticles (NPs) has gained interest due to their application in nanomedicine and biotechnological fields [1]. The NPs can improve drug solubility and stability and increase drug uptake, bioavailability, and efficacy. Nowadays, there are a variety of NPs studied as smart drug delivery systems [2]. NPs can penetrate tissues offering advantages including long circulation time, an improved target-to-non-target concentration ratio, an increased concentration at the target site, and increased cellular internalization due to the EPR (enhanced permeability retention) effect [3,4].

Cyclodextrin chemistry has been exploited in the building of NPs [5,6,7,8]. Cyclodextrins (CyD) are cyclic D-(1)-glucopyranose oligosaccharides that can encapsulate lipophilic drugs and enhance their water solubility, stability, and bioavailability. For this reason, CyDs have been used as excipients in many pharmaceutical preparations [9].

CyDs can form aggregates in water at a millimolar concentration of about 200–300 nm in size, but their applicability in pharmaceutical preparations is very limited by their low physical stability [10].

Various CyD derivatives have been designed for many application fields [11] and for building nanomaterials, spanning from hybrid to organic nanosystems [12,13,14,15,16,17,18]. Great interest is focused on the CyD-based polymers that can form water-soluble and stable NPs [19]. Furthermore, CyD-based NPs have been functionalized with vitamins, sugars, and peptides to actively target cancer cells and increase tumor internalization [15,20,21,22,23,24,25].

Among the targeting biomolecules, hyaluronic acid, or hyaluronan (HA), has been exploited because it possesses many advantages, such as biocompatibility, non-immunogenicity, and possible chemical modification [26,27].

HA is an endogenous polymer of the disaccharide of D-glucuronic acid and N-acetyl-D-glucosamine [28,29]. It is the principal component of the extracellular matrix (ECM) and is involved in different biological processes, such as cell proliferation, cell migration, and regulation of the ECM inflammatory state. The biological activities depend on the molecular weight of the polymer [30]. Most commonly, HA is synthesized as a high-molecular-weight polymer (HMW approximately 1000–8000 kDa). Exogenous HA fragments of low molecular weight (LMW < 200 kDa) have been shown to affect cell behavior through binding to proteins such as CD44 and RHAMM receptors.

In particular, the CD44 receptor belongs to a family of cell adhesion molecules [31]. It is a widely distributed transmembrane glycoprotein that plays a critical role in malignant cell activities, including adhesion, migration, invasion, and survival; it is also strongly implicated in the cell signaling cascades associated with cancer initiation and progression. CD44 is a crucial component in the internalization and metabolism of HA and is endogenously expressed at low levels on various cell types in normal tissues. Tumor-derived cells, however, express CD44 in a high-affinity state that can promote the binding and internalization of HA [31].

HA-based NPs can take advantage of the CD44 receptor-mediated endocytosis uptake in tumor tissues [32,33,34,35]. Among the designed systems, the nanoprodrug HA-paclitaxel conjugate (ONCOFID™-P) is under Phase II clinical trial for the treatment of bladder and ovarian cancers [36].

Recently, CyDs have been functionalized with HA, grafting one or more cavities on HA backbone [37,38,39]. In some conjugates, cross-linked polymers have been synthesized [38,40]. The CyD conjugates have mainly been studied to entrap drugs or biomolecules.

**Figure 1:**
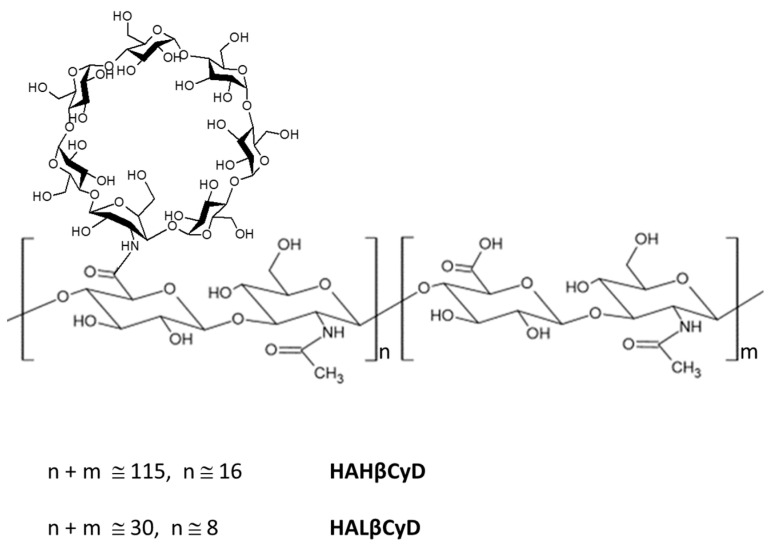
HA β-Cyclodextrin conjugates HAHβCyD and HALβCyD.

Based on the considerable interest in HA derivatives [41], we synthesized new β-CyD grafted HA polymers, starting from HA backbone with two different molecular weights (about 11 kDa and 45 kDa) (Figure 1) under green conditions to compare the two systems in the doxorubicin (DOXO) delivery. DOXO is a powerful anthracycline anticancer drug, widely employed in the treatment of a broad spectrum of cancer types, including breast cancer, Kaposi’s sarcoma, non-Hodgkin’s and Hodgkin’s lymphoma and acute lymphocytic leukemia, neuroblastoma, and bladder cancer despite the severe side effects and emerging multidrug resistance [42]. Many strategies, including nanostructure encapsulation, have been studied to overcome the DOXO side effects and resistance [43]. Among these, Doxil^®^ was the first nanosized (nanoliposome) drug delivery system approved for clinical use in 1995, which showed an improved safety profile and superior efficacy compared to DOXO [44].

Nevertheless, new formulations of DOXO are still of enormous interest [45]. DOXO has also been covalently linked to HA with low efficacy due to the covalent bond [46]. For this reason, we used DOXO as a model drug to investigate the new conjugates as drug carriers.

The new hyaluronan-CyD conjugates can encapsulate DOXO exploiting the properties of CyD and the carboxylate groups of HA. The HA backbone could increase DOXO accumulation in tumor cells by exploiting the CD44-mediated uptake.

Here we characterized the new conjugates HAHβCyD and HALβCyD (NMR and DLS) and tested the affinity for DOXO. We determined the antiproliferative activity of DOXO in the presence of HAHβCyD and HALβCyD at two drug/polymer molar ratios in cancer neuroblastoma cell lines SK-N-SH and SK-N-SH-PMA (overexpressing CD44 receptors).

Neuroblastoma was chosen as a tumor model because DOXO is one of the chemotherapeutics administrated in high-risk neuroblastoma [47].

## 2. Materials and Methods

### 2.1. Materials

All reagents commercially available were used without further purification. 4-(4,6-dimethoxy-1,3,5-triazin-2-yl)-4-methylmorpholinium chloride (DMTMM) and 3A-amino-3 A -deoxy-2 A (S),3A (R)-β cyclodextrin (CyD3NH_2_) were purchased from TCI EUROPE (Zwijndrecht, Belgium). Hyaluronic acids sodium salt (8000–15,000 and 40,000–50,000 Dal) were purchased from Carbosynth (Thal, Switzerland).

Dialysis was carried out with a membrane molecular weight cut-off of 3 KDa (Spectrum Chemical, VWR distributor, Milan, Italy).

Thin Layer Chromatography (TLC) was carried out on silica gel plates (Merck 60-F254). Carbohydrates derivatives were detected on TLC by UV and the anisaldehyde or iodine test.

### 2.2. Synthesis of HAHβCyD

DMTMM (37 mg, 0.1 mmol) and CyD3NH_2_ (126 mg, 0.1 mmol) were added to HA (100 mg, 2 μmol) in 10 mL water in three aliquots (every 30 min). The reaction mixture was stirred at 25 °C for 24 h.

The final product was dialyzed against water.

^1^H NMR: (500 MHz, in D_2_O) δ(ppm):1.9 (s, CH_3_ of N-Acetyl), 3.03–4.00 (m, H-3, -6, -5, -2, -4 of CyDs and HA), 4.17 (m, H-3-A of CyD), 4.36–4.45 (d, H-1 of glucuronic acid and glucosamine), 4.83–5.02 (m, H-1 of CyD).

Size (DLS, Z-average, d): 424 ± 40 nm.

### 2.3. Synthesis of HALβCyD

The synthesis was carried out as reported for HAHβCyD starting from HA (100 mg, 9.1 μmol), DMTMM (126 mg, 0.46 mmol) and CyD3NH_2_ (310 mg, 0.28 mmol).

^1^H NMR: (500 MHz, in D_2_O) δ(ppm): 1.90 (s, CH_3_ of N-Acetyl), 3.20–3.90 (m, H-3, -6, -5, -2, -4 of CyDs and HA), 4.17 (m, H-3-A of CyD), 4.30–4.50 (d, H-1 of glucuronic acid and glucosamine), 4.83–5.02 (m, H-1 of CyD).

^13^C NMR: (125 MHz, D_2_O) δ (ppm): 30.4 (CH_3_), 101.2 (H-1 CyD), 60.4 (C-6 CyD and HA), 70.0–74.0 (C-2, 3, 5, CyD and HA), 80.9 (C-4 CyD); 175.8 (COCH_3_); 178.0 (COOH); 172.0 (NHCO).

Size (DLS, Z-average, d): 176 ± 15 nm.

### 2.4. NMR Spectroscopy

^1^H and ^13^C NMR spectra were recorded at 25 °C with a Varian UNITY PLUS-500 spectrometer at 499.9 and 125.7 MHz respectively. 2D NMR spectra (COSY, TOCSY, HSQC) were performed using 1K data points, 256 increments.

### 2.5. UV-Vis Spectroscopy

UV-Vis spectra were recorded with an Agilent Cary 8500 spectrophotometer equipped with a Peltier cell holder.

### 2.6. Dynamic Light Scattering (DLS)

Dynamic light scattering (DLS) measurements were performed at 25 °C with Zetasizer Nano ZS (Malvern Instruments, London, UK) operating at 633 nm (He–Ne laser). The mean hydrodynamic diameter (d) of the NPs was calculated from the intensity measurement after averaging the five measurements. The samples (1 mg/mL) were diluted in phosphate buffer (pH = 7.4) in ultrapure filtered water (0.2 µm filter).

### 2.7. Experiments of Solubility

DOXO hydrochloride (50 µL, 0.017 M, water solution) was added to 0.200 mL of eight solutions of the CyD polymers in phosphate buffer (100 mM, pH 7.4) at different concentrations as reported elsewhere [32]. The suspensions, formed due to the DOXO precipitation at 7.4 pH, were sonicated for 10 min and incubated at 25 °C in the dark. After 18 h, suspensions were centrifuged at 10,800 rpm for 10 min at 25 °C. The DOXO concentration of the samples was determined in the supernatant with UV/Vis spectroscopy at the wavelength of maximum absorbance (λmax) 482 nm. A linear calibration plot for free DOXO in phosphate buffer at pH 7.4 was previously obtained to obtain the DOXO molar absorptivity ε 10,858 (mol^−1^ L cm^−1^). The CE (complexation efficient) was calculated from the straight-line slope obtained. CE = Slope/(1 − Slope). The apparent stability constant K_11_ = CE/S_0_ was calculated for S_0_ = 3.75 × 10^−5^ M.

### 2.8. MTT (3-(4,5-Dimethylthiazol-2-yl)-2,5-diphenyltetrazolium Bromide) Evaluation of the Antiproliferative Activity of Hyaluronic Acid Complexes

The human cell line SK-N-SH (neuroblastoma) and its derivative, stimulated for seven days with 10 nM phorbol myristate acetate (PMA) in order to allow cells to overexpress CD44 receptor [48], SK-N-SH-PMA were plated in 180 µL into flat-bottomed 96-well microliter plates at 2.22 × 10^4^ cells/mL in complete DMEM added with 10% fetal calf serum (FCS). After 6–8 h, cells were administered with 20 µL containing five concentrations of DOXO alone or in the presence of CyD polymers at 8/1 and 16/1 DOXO/polymer molar ratio diluted in PBS. Plates were then processed as described elsewhere [49].

The compound concentrations inhibiting 50% cell growth (IC_50_) were calculated based on the analysis of the concentration-response curves. Each experiment was repeated 5–7 times.

### 2.9. Immunofluorescence Study of CD44 Expression

SK-N-SH and SK-N-SH-PMA cells were harvested and washed twice with PBS plus 2% FCS. We then pelleted 2.0 × 10^5^ cells that were incubated at 22 °C for 30 min with 50 µL (1:1000) of an anti-CD44 monoclonal antibody (ab254530, Abcam, Cambridge, UK). Cells were then washed twice with phosphate-buffered saline (PBS) plus 2% FCS and incubated again with 50 µL FITC (Fluorescein) AffiniPure F(ab’)2 fragment goat anti-mouse IgG+IgM (H+L) 1:200 dilution (Jackson ImmunoResearch, Ely, UK). After being rewashed twice, cells were evaluated by flow cytometry (Cytoflex-S, Beckman Coulter, Milan, Italy) and analyzed by FlowJo software v10.8 (BD).

### 2.10. Cytofluorimetric Study of Intracellular Accumulation of DOXO and Hyaluronic Acid Complexes

SK-N-SH and SK-N-SH-PMA cells were plated in 96-well plates in 180 µL medium 4 × 10^4^ cells/well. After incubation at 37 °C for 24 h, once reached 75 – 85% confluence, cells were treated with 2 μM DOXO alone or in the presence of CyD polymers at 8/1 and 16/1 DOXO/polymer molar ratio. After 1 h, cells were washed twice with 200 µL of PBS and fixed with 100 µL of 3.7% paraformaldehyde in PBS (containing 2% sucrose) for 15 min [50]. Cells were rewashed with PBS and resuspended in 100 µL PBS containing 2% FCS. Untreated cells were assayed as well.

The intracellular mean fluorescence intensity (MFI) of cells was determined directly in plates by a Glomax Discover microplate reader (Promega Italia, Milan, Italy), using 475 nm excitation and 580–640 nm emission wavelengths. Values were normalized as absolute MFI calculated as MFI of treated cells—MFI of control cells.

### 2.11. Statistical Analysis

Student’s *t* test for independent means was used for the analysis of data.

## 3. Results

### 3.1. Synthesis and Characterization

The synthesis of the HA conjugates with CyD3NH_2_ was carried out in a water solution. DMTMM was used as the activating agent. HA at both molecular weights was obtained in a good yield (50%). The conjugate of HA with the higher molecular weight (45 kDa) was obtained with a lower degree of substitution (DS) (15% of COOH groups) than HA with lower MW (30% of COOH). It has been reported that free HA in water solution forms a duplex secondary structure involving H-bonds between COOH and amido group [51]. The stability of this structure depends on the molecular weight [51]. For this reason, the availability of COOH groups may depend on the HA molecular weight in the condensation reaction in water.

**Figure 2:**
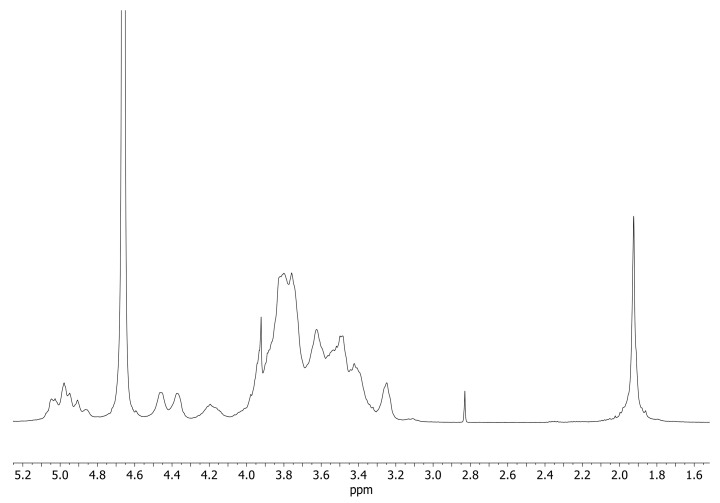
^1^H NMR spectra of HALβCyD (D_2_O, 500 MHz).

^1^H NMR spectra of HAHβCyD and HALβCyD (Figure 2, Figures S1 and S2) show common patterns, H-1 of CyD resonates at about 5 ppm, and the broad signals between 3 and 4 ppm are due to the protons of HA and CyD sugar rings. The peaks observed at 4.3–4.4 ppm are due to the Hs-1 of the glucuronic acid and glucosamine units of HA. The singlet peak at 1.9 ppm corresponds to CH_3_ of the HA N-acetyl group.

The number of CyD grafted to HA polymers was calculated from the ratio between the integral of the CH_3_ signal and CyD Hs-1 (Figures S1 and S2). About 15% of carboxylic groups of HAHβCyD were functionalized with CyD (about 16 units), while 30% of carboxylic groups of HALβCyD were functionalized with CyD units (about 8 units).

### 3.2. Solubility Experiments

**Figure 3:**
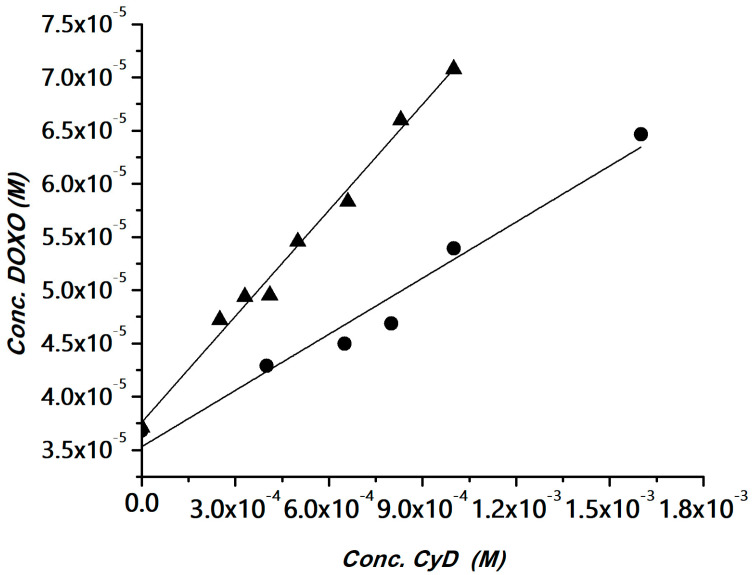
DOXO solubility (phosphate buffer, pH 7.4) versus the amount of HAHβCyD (▲) or HALβCyD (●) (reported as CyD cavity concentration).

The interaction of DOXO with the polymers was investigated using the phase solubility method [52]. The method has been widely used for determining apparent stability constants of drug/CyD complexes in the case of drugs with low water solubility and also for polymeric hosts [53,54,55,56]. The CyD polymers can be considered multi-cavity systems and the cavities can be assumed equivalent and independent binding sites. Solubility experiments were carried out for the HAHβCyD and HALβCyD at pH 7.4 (Figure S3). The solubility phase diagrams were obtained by plotting the DOXO concentration versus the polymer concentrations, expressed as the concentration of CyD units, for a clear comparison among the polymers. Data are reported in Figure 3.

A linear correlation between DOXO solubility and CyD concentration was obtained, with a slope <1 (A_L_ type graph). The diagrams were fitted with the following equation, where CyD is the concentration of the cavities:

S_DOXO_ = So_DOXO_ + [K_11_So/(1 + K_11_So)] × [CyD]

Complexation efficiency (CE) values, calculated from the slope of the solubility diagram, and the apparent stability constant of the CyD unit in the two polymers are reported in Table 1. Data reported in Table 1 showed that CyD in HAHβCyD (DS = 16) is more effective than CyD in HALβCyD (DS = 8). This behavior is in keeping with the general trend reported for CyD grafted to dextrans [57]. It has been reported that in CyD-based polymers that CE is inversely proportional to DS [57]. In fact, for higher DS the cavity becomes less accessible due to steric hindrance of the binding site [57].

The CE and K_11_ values are in the same range as those obtained for other β-CyD systems [24,25,58].

CyD polymers have many binding sites, which we assumed to be identical and independent; the apparent stability constant (K) can be calculated K = nK_11_ (n is the number of CyDs in the polymer) [56]. We found that K is 3840 (M^−1^) for HALβCyD and 15,536 (M^−1^) for HAHβCyD. Carboxylate groups can also contribute to the affinity for DOXO, which is protonated at physiological pH.

### 3.3. Expression of CD44

We stimulated SK-N-SH cells with PMA at a concentration of 10 nM for 7 days to overexpress the CD44 receptors [48]. In these culture conditions, SK-N-SH cells overexpressed CD44 receptors (becoming SK-N-SH-PMA cells) with a relative increase in the percentage of CD44^+^ cells from 53.1 ± 0.7% to 81.0 ± 1.0% (+53%).

The derivative cell line was used to better evaluate the transmembrane passage of DOXO complexes with HAHβCyD and HALβCyD through the CD44 receptor.

### 3.4. Antiproliferative Activity

The antiproliferative activity of DOXO alone and in the presence of HALβCyD and HAHβCyD was studied in SK-N-SH and its derivative SK-N-SH-PMA (Table 2, Figure S4).

Data in Table 2 showed that all complexes had an antiproliferative activity significantly higher than free DOXO in both cell lines, except for DOXO/HAHβCyD and DOXO/HALβCyD complexes 8/1 molar ratio in SK-N-SH, which displayed an antiproliferative activity similar to DOXO. Data suggested that antiproliferative activity depended on the DOXO/polymer molar ratio and the CD44 receptor expression.

SK-N-SH-PMA, which overexpresses CD44 receptors, seems to have a higher sensitivity to the treatment with the DOXO/HAHβCyD and DOXO/HALβCyD complexes 8/1. We hypothesized the CD44 receptor-mediated endocytosis of the complexes, determining a lower IC_50_ in cells overexpressing the CD44 receptor. The lower IC_50_ value for DOXO/HAHβCyD may be due to the higher affinity of HAHβCyD for DOXO.

The highest DOXO/polymer ratio determines a considerable reduction in IC_50_ values (on average reduced by about 70% of free DOXO) and no selectivity for cells overexpressing the CD44 receptor. The highest molar ratio can increase the concentration of the DOXO inclusion complex and improve its uptake in cancer cells.

### 3.5. Intracellular Accumulation of DOXO and Hyaluronic Acid Complexes

In order to establish whether the difference in antiproliferative activity between SK-N-SH and SK-N-SH-PMA treated with DOXO/HAHβCyD and DOXO/HALβCyD was due to a differential DOXO accumulation in target cells, we evaluated DOXO uptake in these cells after administration of DOXO (2 µM) alone or complexed with the CyD polymers.

**Figure 4:**
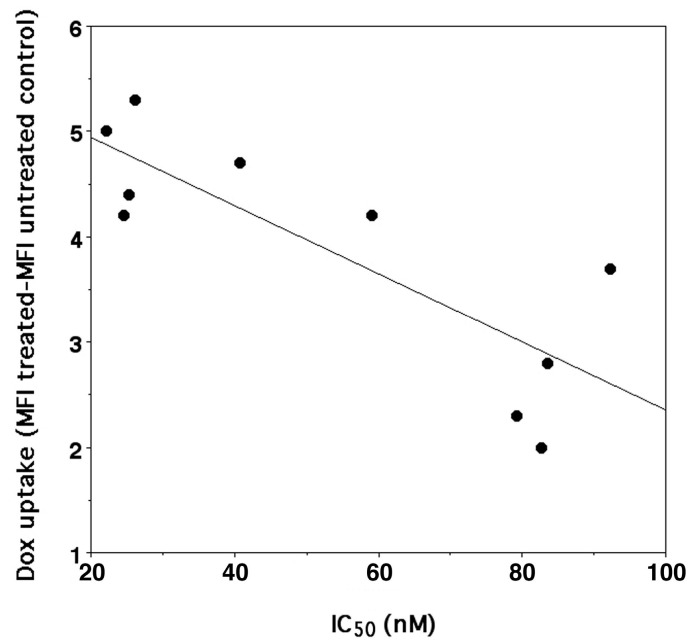
Correlation between IC_50_ of DOXO (y = −0.0323X + 5.5905, *r*^2^ 0.670, *n* = 10, *p* < 0.004) either administered as parent drug or as CyD polymer complexes and its uptake into SK-N-SH and SK-N-SH-PMA cells.

Our data confirmed that, in general, the higher antiproliferative activity correlates (*n* = 10, *r*^2^ = 0.670, *p* < 0.004) with a higher DOXO accumulation in the cells (Figure 4). Furthermore, the higher DOXO uptake was also linked to CD44 overexpression in SK-N-SH-PMA cells but only after treatment with DOXO/HAHβCyD and DOXO/HALβCyD complexes 8/1 (Table 3). This trend may suggest that DOXO complexes at the highest molar ratio studied (16/1) are probably per se sufficient to reach the highest possible antiproliferative activity being able to saturate DOXO molecular targets also in the SK-N-SH cells, which do not overexpress CD44 (only 53% of CD44 receptors).

## 4. Conclusions

We functionalized hyaluronic acid (HA) with two different molecular weights (11 kDa and 45 kDa) with β-cyclodextrin (CyD) to study and compare new conjugates as doxorubicin (DOXO) delivery systems. The conjugates were synthesized in water, unlike other cyclodextrin conjugates synthesized in an organic solvent, with a good conjugation degree.

We found that the CyD grafted to HA with the highest molecular weight showed a better affinity for DOXO due to the higher number of binding sites (16) and a lower degree of substitution (15%).

We tested doxorubicin as a model drug in the presence of two cyclodextrin polymers in neuroblastoma cell lines, SK-N-SH and SK-N-SH-PMA (overexpressing the CD44 receptor). Notably, systems based on hyaluronic acid at the drug/polymer 8/1 ratio improved the antiproliferative activity of doxorubicin selectively in SK-N-SH-PMA with an IC_50_ reduction of up to 56% in the case of CyD grafted to HA at the higher molecular weight. We found that the IC_50_ values correspond to a higher uptake of DOXO in SK-N-SH-PMA (overexpressing CD44), confirming the importance of HA as a target molecule and strongly suggesting the receptor-mediated endocytosis of the complexes. When the drug/polymer 16/1 molar ratio was administrated, the IC_50_ values of DOXO were, on average, reduced by about 70% of free DOXO, although there was no selectivity for SK-N-SH-PMA.

Even if other in vitro and in vivo experiments are needed to explore the capability of the new polymers, the results suggest that they are promising nanoplatforms for doxorubicin delivery.

## Figures and Tables

**Table 1:** CE and apparent stability constant (K) values for the inclusion of DOXO with HAHβCyD and HALβCyD (25 °C, Phosphate buffer, pH 7.4).

Host	CE	K_11_ (M^−1^)	Slope	S_int_
HALβCyD	0.018	480 (± 50)	0.018	3.50 × 10^−5^
HAHβCyD	0.034	971 (± 80)	0.033	3.78 × 10^−5^

**Table 2:** Antiproliferative activity (IC_50_, nM) of DOXO and CyD polymers in SK-N-SH and PMA stimulated SK-N-SH-PMA cells.

Cell Line	DOXO	DOXO/ HALβCyD 8/1	DOXO/ HAHβCyD 8/1	DOXO/ HALβCyD 16/1	DOXO/ HAHβCyD 16/1
SK-N-SH	79.3 ± 23.5	83.5 ± 21.4	92.3 ± 13.4	22.1 ± 9.8 ^a^	24.6 ± 8.5 ^a^
SK-N-SH-PMA	82.7 ± 11.9	58.8 ± 13.4 ^b^	40.7 ± 13.8 ^a,c^	25.3 ± 8.7 ^a^	26.2 ± 3.9 ^a^

^a^*p* < 0.001 vs. DOXO, ^b^ *p* < 0.021 vs. DOXO, ^c^ *p* < 0.001 vs. cells SK-N-SH treated with DOXO/HAHβCyD 8/1.

**Table 3:** Correlation between IC_50_ of complexes DOXO/HALβCyD 8/1 and DOXO/HAHβCyD 8/1 and their DOXO uptake.

Cell Line	MTT DOXO/ HALβCyD 8/1 (IC_50_, nM)	MFI DOXO/ HALβCyD 8/1 ^a^	MTT DOXO/ HAHβCyD/ 8/1 (IC_50_, nM)	MFI DOXO/ HAHβCyD 8/1 ^a^
SK-N-SH	83.5 ± 21.4	2.8 ± 1.6	92.3 ± 13.4	3.7 ± 0.7
SK-N-SH-PMA	58.8 ± 13.4 ^b^	4.2 ± 1.0	40.7 ± 13.8 ^c^	4.8 ± 2.0

^a^ Values were normalized as absolute MFI calculated as MFI of treated cells—MFI of control cells. ^b^ *p* = 0.0536 vs. SK-N-SH cells, as evaluated by the Student’s *t*-test for independent means.^c^ *p* = 0.0008 vs. SK-N-SH cells, as evaluated by the Student’s *t*-test for independent means.

## Data Availability

Data will be made available on request.

## Supplementary Materials

The following supporting information can be downloaded at: https://www.mdpi.com/article/10.3390/pharmaceutics15020374/s1, Figure S1: ^1^H NMR spectrum of HALβCyD; Figure S2: ^1^H NMR spectrum of HAHβCyD; Figure S3: UV-Vis spectra of saturated solution of DOXO in the presence of an increasing concentration of the polymer; Figure S4. Dose-response curves of SK-N-SH (top) and SK-N-SH-PMA (bottom) cells treated with DOXO, DOXO/HALβCyD, or DOXO/HAHβCyD.
