# Supplementary material for: Hyaluronan-Cyclodextrin Conjugates as Doxorubicin Delivery Systems
Source: Pharmaceutics. 2023 Jan 21;15(2):374. doi: 10.3390/pharmaceutics15020374 (PMC9963997; doi:10.3390/pharmaceutics15020374)
Supplement: Supplementary file 1 [file pharmaceutics-15-00374-s001.zip › pharmaceutics-2142098-supplementary.pdf]

Hyaluronan-Cyclodextrin Conjugates as Doxorubicin Delivery Systems

Noemi Bognanni [1], Maurizio Viale [2], Luana La Piana [1], Simone Strano [1], Rosaria Gangemi [2], Cinzia Lombardo [3], Maria Teresa Cambria [3] and Graziella Vecchio [1,*]

[1] Dipartimento di Scienze Chimiche, Università degli Studi di Catania, Viale A. Doria 6, 95125 Catania, Italy
[2] UOC Bioterapie, IRCCS Ospedale Policlinico San Martino, Largo R. Benzi 10, 16132 Genova, Italy
[3] Dipartimento di Scienze Biomediche e Biotecnologiche, Sezione di Biochimica Medica, Università degli Studi di Catania, Via S. Sofia 97, 95125 Catania, Italy
* Correspondence: gr.vecchio@unict.it

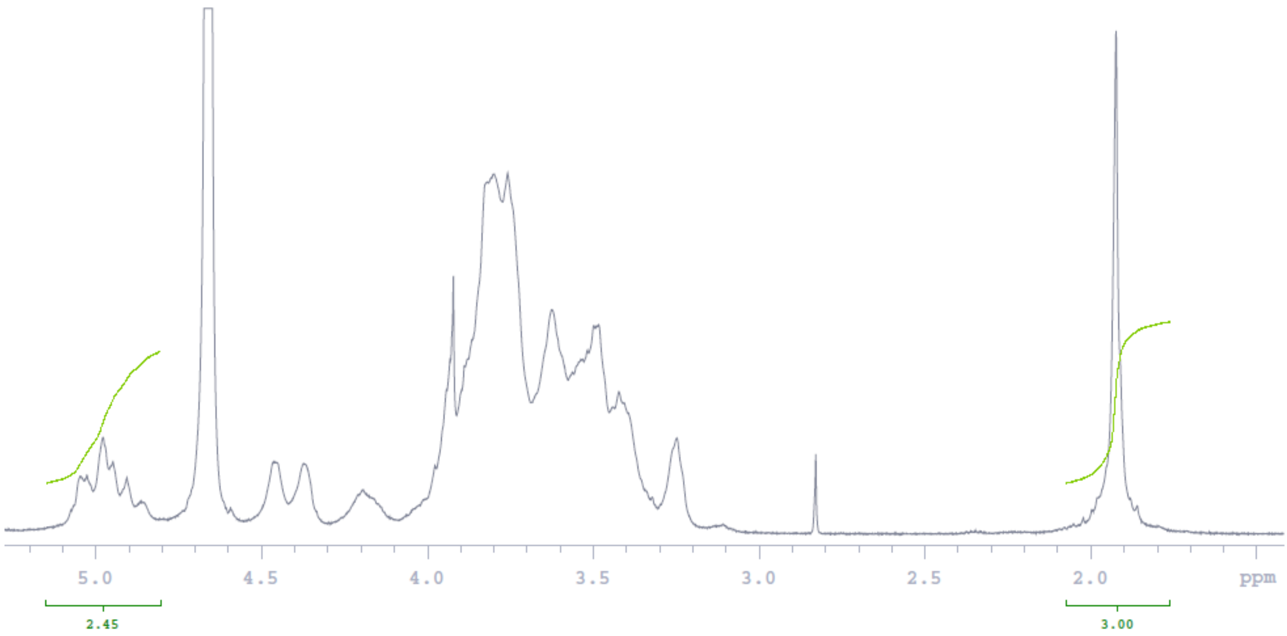

Figure S1. ^{1}H NMR spectrum of HALβCyD (D_2O, 500 MHz)

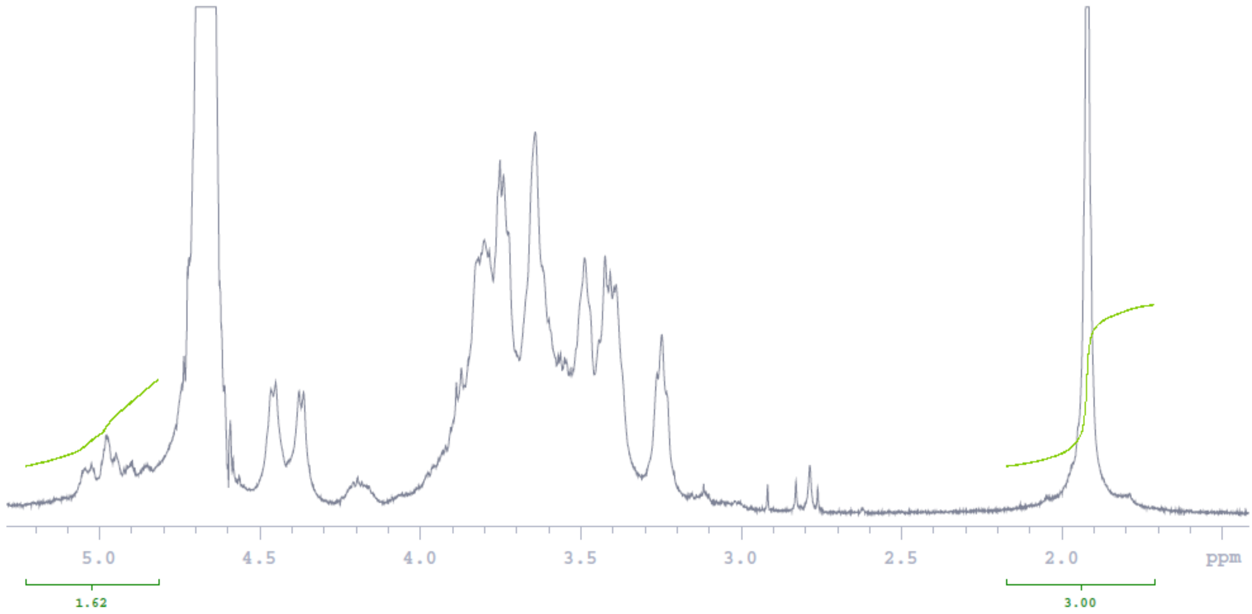

Figure S2. ^{1}H NMR spectrum of HAHβCyD (D_2O, 500 MHz)

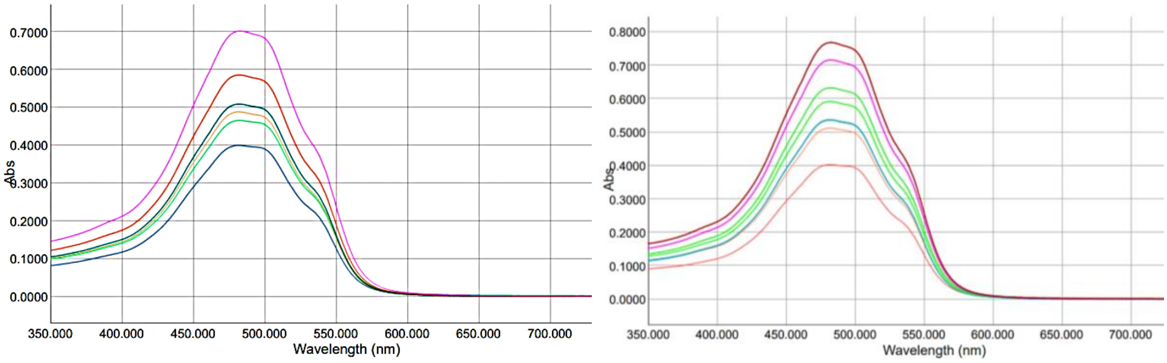

Figure S3. UV-Vis spectra of saturated solution of DOXO in the presence of an increasing concentration of the polymer: HALβCyD (Left) and HAHβCyD (right) at pH 7.4, phosphate buffer

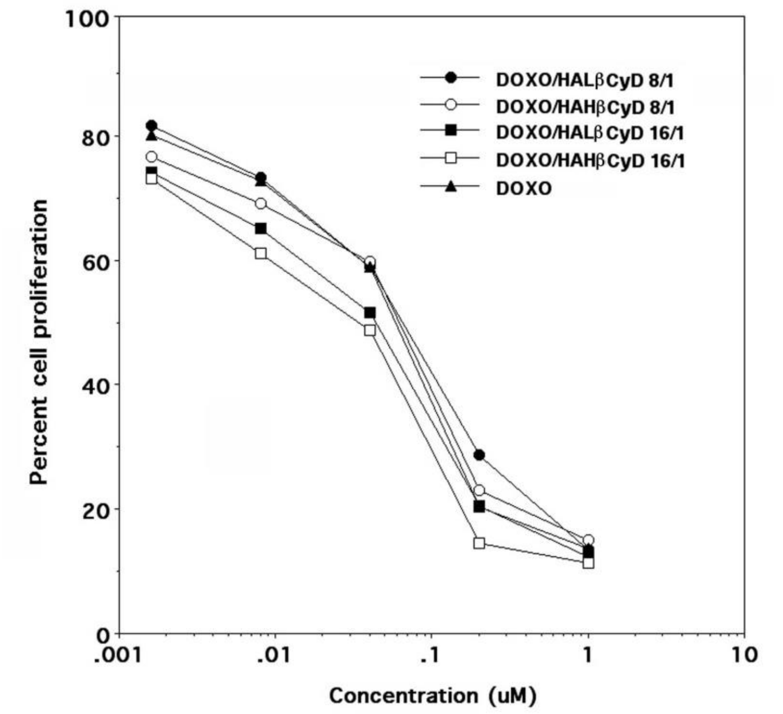

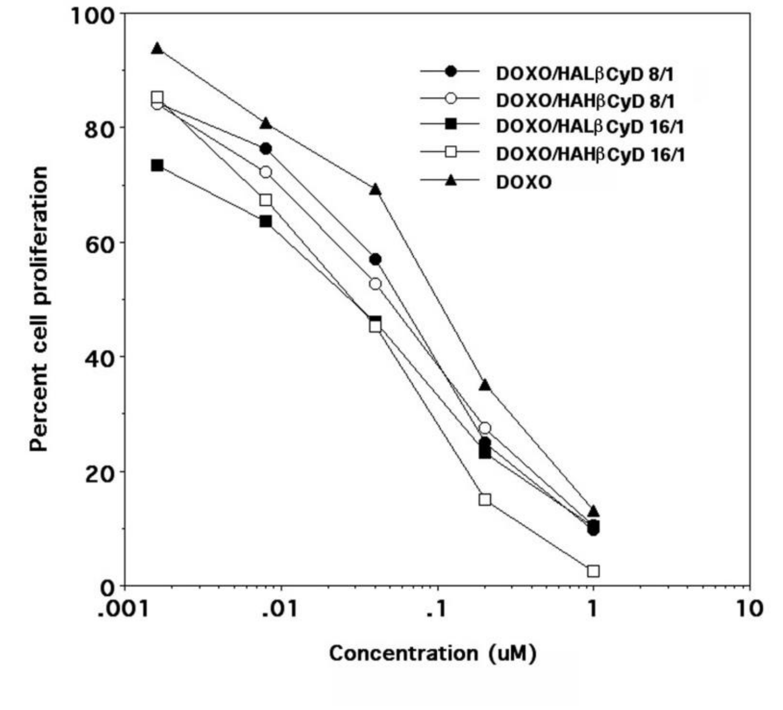

Figure S4. Graphs represent the mean dose-response curves of SK-N-SH (top) and SK-N-SH-PMA (bottom) cells treated with DOXO or DOXO/HALβCyD 8/1, DOXO/HAHβCyD 8/1, DOXO/HALβCyD 16/1, and DOXO/HAHβCyD 16/1 complexes.
